# Two sides to colon cancer: mice mimic human anatomical region disparity in colon cancer development and progression

**DOI:** 10.20517/2394-4722.2018.39

**Published:** 2018-09-27

**Authors:** Jessica Felton, Kunrong Cheng, Aaron C. Shang, Shien Hu, Shannon M. Larabee, Cinthia B. Drachenberg, Jean-Pierre Raufman

**Affiliations:** 1 Department of Surgery, University of Maryland School of Medicine, Baltimore, MD 21201, USA.; 2 Department of Medicine, University of Maryland School of Medicine, Baltimore, MD 21201, USA.; 3 Department of Pathology, University of Maryland School of Medicine, Baltimore, MD 21201, USA.

**Keywords:** Colorectal cancer, orthotopic tumor model, mouse model, HT-29 cells, colon

## Abstract

**Aim:**

Strong evidence reveals important differences between cancers in the proximal *vs*. distal colon. Animal models of metastatic colon cancer are available but with varying degrees of reproducibility and several important limitations. We explored whether there were regional differences in the location of murine colon cancers and assessed the utility of murine models to explore the biological basis for such differences.

**Methods:**

We re-analyzed data from our previous studies to assess the regional distribution of murine colon cancer. In survival surgery experiments, we injected HT-29 human colon cancer cells into the wall of the cecum or distal colon of Nu(NCr)-Foxn1^nu^ or NOD.Cg-Prkdc^scid^Il2rg^Tim1Wji^/SzJ mice and compared the development of primary tumors and metastases.

**Results:**

Within 7–17 weeks after intramural cecal injection of HT-29 cells, eight mice failed to develop solid primary tumors or metastases. In contrast, within four weeks after cell injection into the distal colon, 13 mice developed metastases - 12 mice developed subcutaneous metastases; of these, four developed liver metastases and one developed both liver and lung metastases. One mouse developed liver metastases only. Histological examination confirmed these lesions were adenocarcinomas.

**Conclusion:**

Our findings reveal the preferential growth of murine colon neoplasia and invasive human orthotopic xenografts in the distal mouse colon. The new approach of injecting cells into the distal colon wall results in a pattern of colon cancer development that closely mimics the progression of metastatic colon cancer in humans. This novel model of colon neoplasia has great potential for exploring anatomical differences in colon cancer and testing novel therapeutics.

## INTRODUCTION

In developed countries, colorectal cancer (CRC) is the second most common cause of cancer-related death in men and the third in women^[1]^. Metastatic cancer is the chief reason for CRC-related death; primary tumors without metastases are readily cured by endoscopic or surgical therapy^[1]^. Intriguingly, strong evidence reveals important differences between cancers in the proximal *vs.* distal colon^[2–6]^.

Compared to cancers of the distal colon, proximal colon cancers are more common in women, are associated with microsatellite instability and the serrated pathway, and are more likely to be at advanced stages when first diagnosed. Distal colon tumors are more likely to be associated with chromosomal instability and arise from the pathway involving dysregulated *APC*, *K-ras*, *DCC*, and *p53*^[6]^. Previous studies reported conflicting findings with regards to whether mortality was significantly different in those with primary right- *vs.* left-sided colon cancer^[7–9]^. A meta-analysis found higher mortality in patients with right-sided compared to left-sided colon cancer^[7]^. A recent database study found that right-sided colon cancer was associated with lower cancer-specific mortality at the localization stage, equivalent mortality at the regional stage, and higher mortality at the metastatic stage^[7]^. Another recent retrospective study found those with left-sided colon cancer had better survival outcomes, especially with stage III cancers^[10]^. From 1998 to 2013, the SEER (Surveillance, Epidemiology, and End Results) database identified 90,635 and 112,679 persons diagnosed with left- and right-sided colon cancer, respectively^[7]^.

Few therapeutics are either effective or available to treat persons with metastases to the liver and other organs. To improve therapeutic outcomes, there is great urgency to gain a better understanding of the mechanisms underlying colon cancer dissemination as a basis to develop targeted therapies. For investigators to test such new therapeutics with some degree of reliably there is also a great need to conceive and develop novel models that more closely mimic human disease.

Several animal models of metastatic colon cancer are available, with varying degrees of reproducibility, limitations, and imperfect fidelity to the biology of human cancer. Current murine models are limited by location, depending on what model is used, and cancers in different locations have different genetic profiles. A case in point is ApcMin mouse models that were meant to recapitulate defective Wnt/β-catenin signaling present in ~90% of human colon cancer^[11]^; in the most commonly used ApcMin mouse strains, tumors are almost uniformly adenomas, not adenocarcinomas, and are located predominantly in the small intestine, not colon. Also, murine models using injection of human colon cancer cells are limited by the need to use immune-deficient mice to allow tumors to develop, thus excluding the testing of immunotherapies^[12]^. Nonetheless, using syngeneic models with murine colon cancer cells is also imperfect because these cell lines are less well-studied and their biology may not mimic that of human cancers^[12]^.

Despite their limitations, murine models have long served as the most reliable platform for preclinical evaluation of new drugs and technologies^[12]^. These include models employing chemical carcinogenesis, genetic engineering, and animal- or patient-derived xenografts^[1,13]^; the latter have been particularly helpful to study the mechanisms underlying the metastatic spread of human colon cancer and identify susceptible therapeutic targets^[1,12]^. Colorectal cancer xenografts grown subcutaneously in immunodeficient mice are limited by the lack of metastasis; instead, orthotopic tumor models involving injection of CRC tumor cells or implantation of tumor tissue directly into the wall of the colon have the potential to be more representative of human metastasis^[14]^.

We sought to determine if mice could serve as a model to explore regional differences in the location of cancers within the colon. To seek such differences in the growth and progression of colon neoplasia in mouse models, we first determined the location of colon tumors in mice treated with a colon-selective carcinogen or with a genetic predisposition to intestinal neoplasia. Next, based on our initial findings, we compared the proclivity of human colon cancer cells to grow and invade the proximal *vs.* distal colon of immune-deficient mice.

## METHODS

### Analysis of the distribution of colon neoplasia in our published studies of chemically-induced carcinogenesis in mice

To assess the regional distribution of murine colon cancer, we re-analyzed data from our published and unpublished murine colon cancer studies conducted from 2006 through 2018^[15–19]^. During this interval, we had treated 10- to 23-week-old male mice on a variety of genetic backgrounds with weekly intraperitoneal injections of 7.5 mg azoxymethane (AOM)/kg body weight for 4 weeks. In C57BL/6 mice that are resistant to AOM treatment alone^[20]^, we supplemented the drinking water with 2.5% dextran sodium sulfate (DSS) for 5 days. We euthanized mice 20 weeks after the first AOM injection. An investigator masked to mouse genotype and treatments measured tumor number and size, and tumors were characterized as adenomas or adenocarcinomas based on size, contour, and color. A senior pathologist classified colon tumors as adenomas or adenocarcinoma based on consensus recommendations^[21]^.

### Surgical induction of colon neoplasia

#### Cell culture

We purchased authenticated HT-29 cells from American type culture collection (ATCC). HT-29 cells were grown in McCoy’s 5A medium (Life Technologies) supplemented with 10% FBS. We grew cells in a humidified incubator at 37 °C with 5% CO_2_ and passaged weekly at subconfluence after trypsinization. We suspended cells in DPBS (50 × 10^6^ cell/mL) containing 10 μmol/L Y27632 and 50% Matrigel.

#### Animals

All animal studies were conducted at the Baltimore VA Hospital Animal Facility and our laboratory in the Bressler Research Building at the University of Maryland School of Medicine. All surgical procedures were approved by the University of Maryland School of Medicine Institutional Animal Care and Use Committee under the Office of Animal Welfare Assurance. The Research and Development Committee at the Baltimore VA also approved animal studies. We used 11- to 14-week old male Nu(NCr)-Foxn1^nu^ (nude) mice and NOD. Cg-Prkdc^scid^Il2rg^Tim1Wji^/SzJ (NSG) mice, obtained from both the University of Maryland Veterinary Resources and Jackson Laboratories (Bar Harbor, ME).

#### Surgical technique - laparotomy

In a biosafety cabinet (BSL2) we anesthetized mice with continuous vaporized isoflurane for general anesthesia and performed laparotomy and cell injections with the mice on a warming pad. After confirming a sufficient level of anesthesia by a toe pinch, we positioned mice prone. For corneal protection, we applied lubricant (Major Pharmaceuticals LubriFresh P.M Ophthalmic Ointment, Livonia, MI) to each eye. We dis-infected the mouse’s upper back with an alcohol swab and administered buprenorphine SR (concentration 0.3 mg/mL, dose 0.05–0.1 mg/kg body weight diluted 1:9 with sterile 0.9% saline) or carprofen (concentration 50 mg/mL, dose 5 mg/kg body weight diluted 1:9 with sterile 0.9% saline) subcutaneously for analgesia. We then placed mice supine and, if necessary, clipped the anterior abdominal hair. After skin preparation with alcohol, to provide local anesthesia we injected mice subcutaneously with 0.25% bupivacaine (concentration 2.5 mg/mL, dose 0.1 mL diluted 1:2 with sterile 0.9% saline) along the planned midline laparotomy site. Next, we cleansed the abdomen with povidine-iodine solution and alcohol and applied sterile drapes. We made a small midline laparotomy and inserted a self-retaining retractor in the upper abdomen.

Following injection of human colon cancer cells and replacing the intestines, we approximated fascial edges with 5–0 vicryl running sutures and closed the skin primarily with 4–0 nylon interrupted sutures. After applying skin glue (3M Vetbond tissue adhesive, St. Paul, MN) to the suture line, we awakened mice slowly from anesthesia and placed them in a clean cage for recovery with close monitoring. After completion of the operation, the mice were administered analgesia for at least 72 h post-operatively and monitored closely.

#### Surgical technique - cecal injection

To explore the predilection of human colon cancer cells to form tumors in different regions of the mouse, we first injected 2 to 5 × 10^6^ (40–100 μL) HT-29 human colon cancer cells into the cecum of nude or NSG mice. We chose these cell numbers based on previous reports describing successful metastatic models of colon cancer in mice^[1,12,22,23]^. We described pre-operative steps above. The cecum was located using moist sterile cotton tip applicators and brought outside the abdomen onto a moist 2 × 2 sterile gauze. In all mice, we injected 2–5 × 10^6^ cells (40–100 μL) into the wall of the cecum using a 27-guage needle. After injection, we applied light pressure at the injection site for approximately 30 s with a moist sterile tip applicator and inspected the area for leakage. We irrigated the cecum and abdominal cavity with warm DPBS, and then returned the cecum to its normal anatomic position within the abdomen. Closure of the abdomen was performed as describe above.

#### Surgical technique - flank injection

In mice failing to form cecal tumors, we confirmed the ability of the HT-29 cells to form xenografts and metastases following subcutaneous and splenic injection, respectively. For subcutaneous injections, we briefly anesthetized mice with vaporized isoflurane, disinfected their flanks with alcohol, and injected 2 × 10^6^ cells (40 μL) in each flank. We recovered mice from anesthesia in their cages.

#### Surgical technique - splenic injection

We described pre-operative steps above. The spleen was located using moist sterile cotton tip applicators and brought forward within the abdomen. In all mice, we injected 5 × 10^6^ cells (100 μL) into the wall of the spleen using a 27-guage needle. After injection, we applied light pressure at the injection site for approximately 30 s with a moist sterile tip applicator and inspected the area for leakage and bleeding. We irrigated the spleen and abdominal cavity with warm DPBS, and then returned the spleen to its normal anatomic position within the abdomen. After 1 h, we removed the spleen and irrigated the abdomen again with DPBS. We closed the abdomen as described above.

#### Surgical technique - distal colon injection

**Figure 1.**
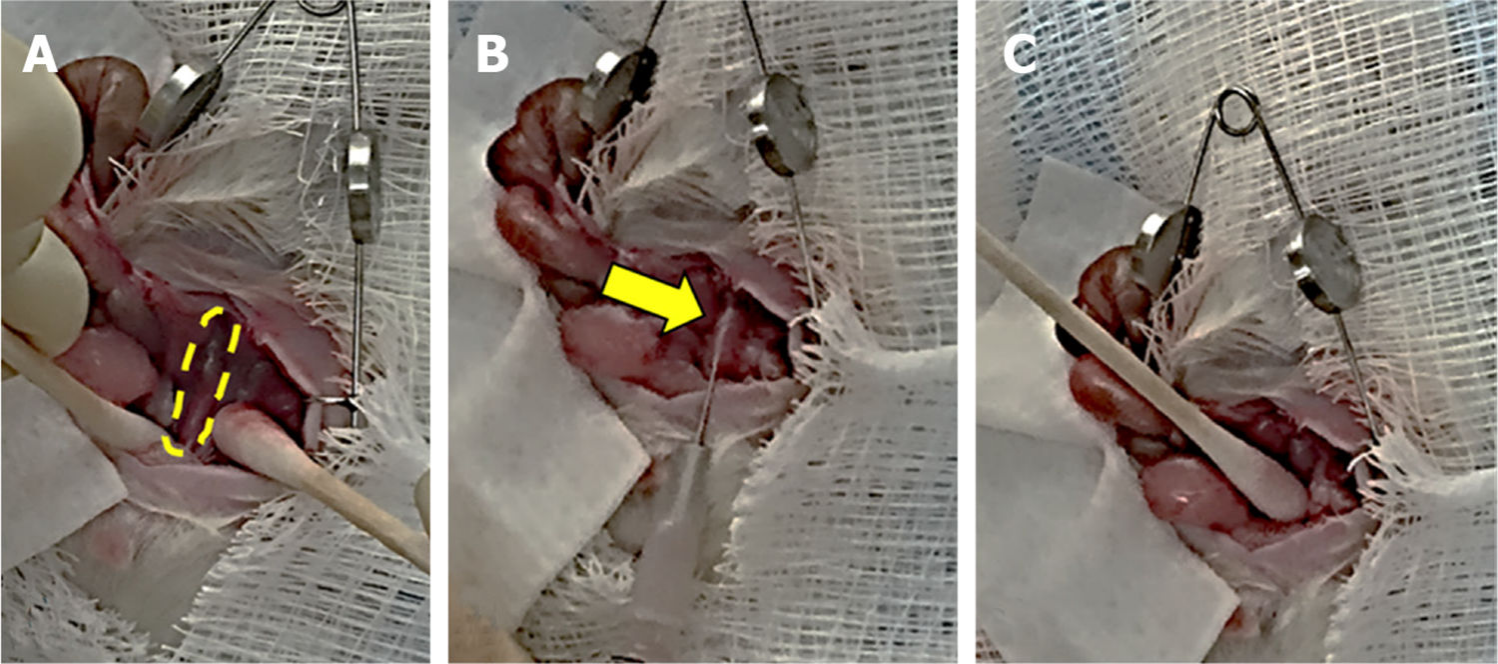
Main steps in the surgical approach to injecting colon cancer cells in the murine distal colon. A: Isolation of the distal colon (outlined) using moist sterile cotton tip applicators with retraction of the abdominal wall and evisceration of abdominal organs; B: injection of 5 × 10^6^ HT-29 human colon cancer cells into the wall of the distal colon (arrow) using a 27-guage needle; C: applying pressure with a moist sterile cotton tip applicator at the injection site to prevent leakage and hemorrhage

To induce colon cancer growth and metastasis, we injected 5 × 10^6^ (100 μL) HT-29 human colon cancer cells into the wall of the distal colon of nude or NSG mice. We described pre-operative steps above. The distal colon was located using moist sterile cotton tip applicators [Figure 1A]. In all mice, we injected 5 × 10^6^ cells (100 μL) into the wall of the distal colon using a 27-guage needle [Figure 1B]. After injection, we applied light pressure at the injection site for approximately 30 s with a moist sterile tip applicator [Figure 1C] and inspected the area for leakage. We irrigated the distal colon and abdominal cavity with warm DPBS. We closed the abdomen as described above.

### Statistical analysis

We used the unpaired Student’s *t* test (assuming unequal variance) to compare continuous variables between two independent groups. For multi-group comparisons, we applied two-way ANOVA with one between-subject factor (WT *vs.* FGF15-deficient) and one within-subject factor (normal tissue *vs.* tumor tissue) followed by post hoc tests with Tukey-Kramer’s adjustment for *P* values. We used Fisher’s exact test to compare proportions. We considered differences significant when P was less than 0.05.

## RESULTS

### Chemical induction of colon cancer

During a 12-year period, we induced colon neoplasia by treating 182 mice with AOM alone and 94 mice with AOM plus DSS. Strikingly, in all AOM- and AOM/DSS-treated mice that developed adenomas and adenocarcinomas [265 of 276 mice (96%)], all tumors were limited to the distal half of the colon; no proximal lesions were present (*P* < 0.001). None of the 276 mice that developed primary colon tumors had metastases.

### Surgical induction of colon cancer

#### Cecal/flank/splenic injection

**Figure 2.**
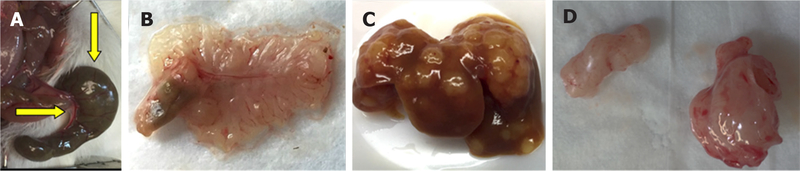
Results of cecal, splenic, and subcutaneous injections of HT-29 human colon cancer cells. A, B: Serosal and mucosal images of normal cecum 15 weeks after injecting HT-29 cells; C: numerous liver metastases 4 weeks after splenic injection; D: representative xenografts harvested from mouse flanks 4 weeks after subcutaneous flank injection

Seven to 17 weeks after cecal injection of HT-29 cells, none of 8 mice (5 nude, 3 NSG) developed a cecal lesion [Figure 2A and B]. Yet, within 4 weeks, injecting HT-29 cells into the spleens and flanks of nude mice (3 mice each) uniformly yielded liver metastases [Figure 2C] and xenografts [Figure 2D], respectively, confirming the cells were capable of developing solid tumors that grew and metastasized. No metastases developed after flank injection and xenograft formation.

#### Distal colon injection

**Figure 3.**
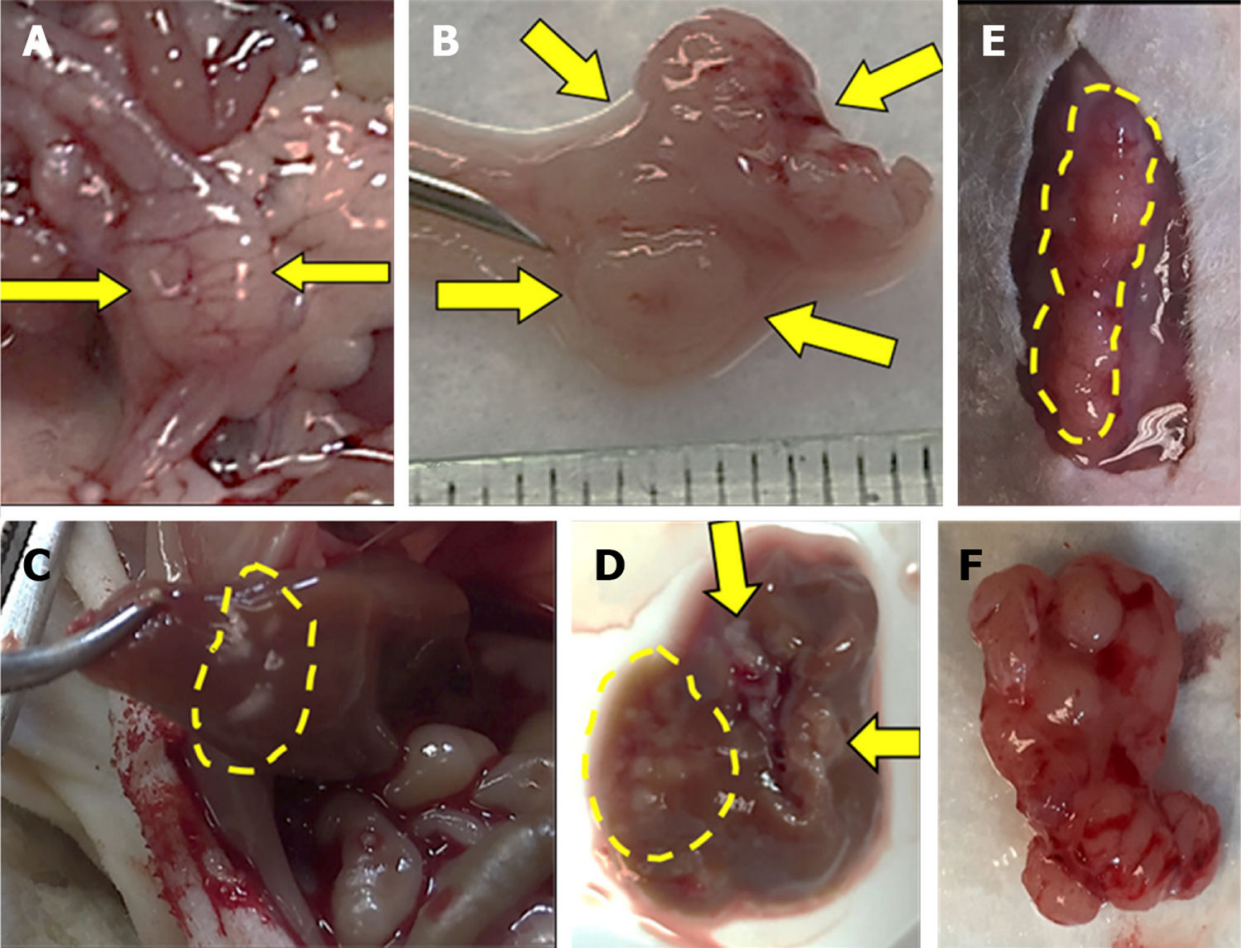
Injected human colon cancer cells form solid tumors in the distal colon with liver, lung, and anterior abdominal wall metastases. Serosal (A) and mucosal (B) images show invasive solid tumor in the distal colon (arrows). Metastases in the liver, *in situ* (C) and *ex vivo* (D) (arrows and dashed lines). Subcutaneous metastases in the anterior abdominal wall, in situ (E) and *ex vivo* (F).

**Figure 4.**
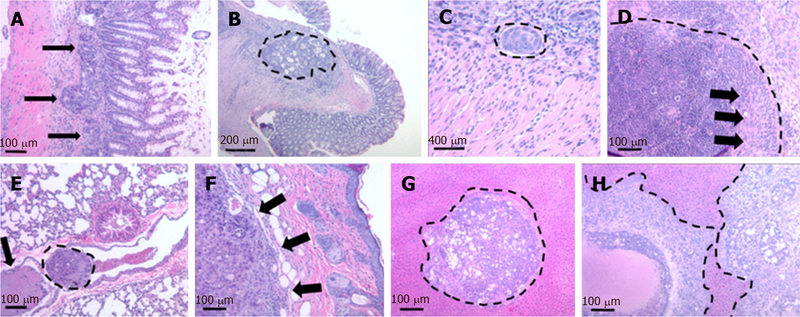
Representative histological images of local colon tumor as well as metastases to the lung, liver, and anterior abdominal wall. (A) and (B) Primary tumor invading the intestinal wall (arrows and dashed lines); C: tumor emboli in intramural and subserosal lymphatics (dashed lines); D: lymph node infiltration. Dashed lines delineate lymph node capsule, arrows indicate tumor cells; E: metastasis to the lung: intravascular tumor embolus (dashed lines) and thrombus (arrow); F: subcutaneous metastasis to anterior abdominal wall (arrows) with epidermis to the right; G and H: multiple metastatic tumor deposits within the liver (dashed lines).

Within four weeks after cell injection, 12 mice developed primary colon tumor at the injection site in the distal colon and 13 mice (4 NSG, 9 nude) developed metastases [Table 1]. Based on our preliminary mouse experiments as well as the results of previously published studies^[1,23,24]^, we euthanized mice four weeks after cell injection. Twelve mice developed subcutaneous anterior abdominal metastases; of these, four developed liver metastases and one developed both liver and lung metastases [Figure 3]. One mouse developed liver metastases only. Histological examination confirmed the presence of adenocarcinoma within the wall of the mouse colon [Figures 4A and B], in the lymphatic spaces [Figures 4C and D], in the lung parenchyma [Figure 4E], in the anterior abdominal wall [Figure 4F], and multiple metastatic deposits within the liver [Figures 4G and H].

## DISCUSSION

Increasing evidence supports the presence of major differences in right- and left-sided colon cancers with regard to the host’s clinical characteristics, microbiome, response to treatment and outcome. Although molecular and genetic profiling of cancer cells has revealed some important differences, the reasons for this anatomical disparity remain unclear^[25,26]^. Whether the primary tumor is located in the right or left colon has been reported to play a prognostic role in metastatic colorectal cancer^[25]^, although new findings suggest primary tumor ‘sidedness’ may be a less important determinant of overall and disease-specific survival than patient characteristics and other pathological features^[26]^.

Our studies reveal that mice appear to be a suitable model to explore the predilection of cancer for different anatomical regions of the colon. We found that mice treated with a colon-selective carcinogen or with a genetic predisposition to intestinal neoplasia developed tumors only in the distal half of the colon; there were no proximal lesions. Likewise, immune-deficient mice preferentially developed colon cancer and metastases when we injected HT-29 human colon cancer cells into the distal, rather than proximal, colon.

In the course of these studies, we developed a novel method for inducing metastatic colon cancer in mice. We chose HT-29 cells for our studies because they a are commonly used *in vitro* model of human colon cancer and express M3 type muscarinic receptors (M3R), a focus of our research program^[27–29]^. Injecting HT-29 human colon cancer cells between the mucosa and the muscularis external layers of the distal colon wall of immunodeficient mice resulted in a pattern of tumor dissemination that mimicked human disease. Nonetheless, while it is uncommon for colon cancer in humans to spread to the skin and subcutaneous tissues, we found that when injected in mice, HT-29 cells are capable of diffuse metastasis, including to the skin. At present, we cannot explain why these colon cancer cells had a predilection for the anterior abdominal subcutaneous tissue. The location of these skin metastases near the surgical incision site leads us to speculate that features of the inflammatory response to the skin incision (e.g., release of cytokines) may attract migrating colon cancer cells to this location, a testable hypothesis that may expand our understanding of the biology underlying tumor metastasis. We will test this hypothesis in future studies.

We believe this novel animal model will be an important adjunct to our *in vitro* studies and useful to study and test novel therapies that target M3R and its downstream signaling pathways to attenuate cancer cell dissemination. This model is relatively straightforward and the procedures easy to learn and perform by an investigator experienced in animal surgery, with reasonably rapid development of primary solid tumors and metastases. Unlike xenograft models, this method requires only one mouse and one operation to generate both colon cancer and metastasis. Our approach appears more biologically relevant than models in which investigators inject cells into the tail vein or footpad.

An important limitation of our new approach is that, as discussed in the Introduction, colon cancer immunotherapy cannot be studied in models using immune-deficient mice. However, humanizing the mouse immune system may achieve this goal. Next-generation models, including “immunoavatar mice” could offer the ability to study the effects of immunotherapy in colon cancer. Hemato-lymphoid humanized mouse models may allow the development of a complete human immune system in a human tumor-bearing mouse^[30]^. Yet, even these humanized models are likely to present important obstacles with regard to mimicking the physiological maturation of human immune cells and the progression of human colon cancer.

In conclusion, our findings identify preferential growth of murine colon neoplasia and invasive human orthotopic xenografts in the distal mouse colon. These data support the utility of mouse models to study anatomical variance in the development and progression of colon neoplasia. We describe a useful model for inducing metastatic colon cancer in mice that is neither laborious nor time-consuming. This novel approach furnishes animals that closely mimic the progression of metastatic colon cancer in humans. This approach shows promise for studying novel therapeutics targeting colon cancer dissemination and metastasis.

## Figures and Tables

**Table 1.** Distribution of primary colon tumors and metastases after injection of human colon cancer cells into the distal colon wall of 13 mice

Mouse	Strain	Primary colon tumor	Liver metastases	Abdominal wall metastases	Lung metastases
1	NSG	√	-	√	-
2	NSG	√	-	√	-
3	NSG	√	-	√	-
4	NSG	√	-	√	-
5	Nude	√	-	√	-
6	Nude	√	√	√	-
7	Nude	√	√	√	√
8	Nude	-	-	√	-
9	Nude	√	-	√	-
10	Nude	√	√	√	-
11	Nude	√	√	√	-
12	Nude	√	√	√	-
13	Nude	√	√	-	-

NSG: NOD.Cg-Prkdc^scid^Il2rg^Tim1Wji^/SzJ.
